# An analysis of cost-saving in the German statutory health insurance system due to the introduction of epoetin alfa biosimilars in Germany

**DOI:** 10.3389/fpubh.2025.1667315

**Published:** 2025-12-16

**Authors:** Ilka Raitner, Barbara Müller, Anjaly Vijayan, Lena Zimmermann, Peter Bramlage, Nils-Henning Ness, Markus Müller

**Affiliations:** 1 Sandoz Deutschland/Hexal AG, Holzkirchen, Germany; 2 Institute for Pharmacology and Preventive Medicine, Cloppenburg, Germany

**Keywords:** biosimilars, epoetin alfa, cost-savings, Germany, German statutory health system

## Abstract

**Aims:**

Epoetin alfa, an erythropoiesis-stimulating agent, is the mainstay for treating severe anemia caused by kidney disease or induced by cancer-chemotherapy. This study aims to investigate the potential for reducing drug costs through the utilization of epoetin alfa biosimilars within the German statutory health insurance system (SHI).

**Methods:**

For the analysis, price and product data from ABDATA with sales volume data from IQVIA Pharmascope for the period from 2008 to 2023 were pooled. This enabled a comparison of cost savings due to the market introduction of epoetin alfa biosimilars from 2007 onwards.

**Results:**

From 2008 to 2023, overall epoetin alfa unit volume has increased by a total of 23%. Compared to the other epoetin derivatives such as, epoetin beta, zeta and theta, an average unit share of 57% (between 2008 and 2023) highlights the importance of epoetin alfa within the segment of erythropoiesis-stimulating agents. Among the various dosing strengths of epoetin alfa, the nephrological strength 4,000 IU formulation has gained notable sales growth (12%) from 2008 to 2023. Economically, the introduction of epoetin alfa biosimilars has led to substantial cost-savings for the SHI, amounting to €505,731,202 between 2008 and 2023 for nephrological strengths. Due to biosimilar competition, the price of epoetin alfa decreased by 47% from 2007 to 2012, further contributing to the diminished price difference between originator epoetin and other epoetin alfa biosimilars. Notably, the 4,000 IU nephrological strength alone accounted for savings of €143,720,363, reflecting its widespread clinical adoption.

**Conclusion:**

The introduction of epoetin alfa biosimilars has not only enhanced clinical accessibility, but also yielded significant cost savings for the German healthcare system. This underscores the dual role of biosimilars in improving healthcare affordability and maintaining effective therapeutic outcomes.

## 1 Introduction

Pharmaceutical prices in Germany are initially determined freely by the originator manufacturer (1). Following regulatory approval, prescription medicines can typically be launched directly onto the German market. Thereby, patients insured under the statutory health insurance (SHI) scheme, a mandatory, universal healthcare system which includes about 90% of all German citizens, are entitled to reimbursement of prescription medicine costs (1). The SHI funds operate on the principle of solidarity, with contributions determined according to each insured individual’s financial capacity, while the scope of insurance benefits remain identical for all members (2). Since 2011, all newly marketed drugs undergo an early benefit assessment within the first year after launch, including negotiations on the reimbursed price (3). Once market exclusivity and patent protection expire, generic or biosimilar products may enter the market, which is expected to generate substantial cost savings for health insurers (4). The entry of such follow-on products also triggers price competition, shaped by specific regulatory mechanisms but primarily driven by direct market forces (4, 5).

Several price regulation policy instruments are available to foster competition in the German pharmaceutical market, especially after loss of exclusivity. Health insurers may enter into rebate contracts with pharmaceutical companies, enabling preferential dispensing of specific products in pharmacies (4). Competitive list prices also provide advantages in market positioning. Furthermore, the establishment of reference price systems allows maximum reimbursement thresholds to be set for defined therapeutic classes or specific molecules, thereby exerting additional downward pressure on prices (4, 5). These thresholds may be further adjusted with intensified price competition within the therapeutic class (6). Despite the fact that biologic drugs are – up until 2025 – in most cases not part of an automatic substitution at pharmacy level, above mentioned pricing regulation instruments apply for all prescription medicine, both small molecule and biologic drugs.

In this regulatory and economic environment, biosimilars have become an increasingly important instrument for cost containment. Biosimilars are biological products that are highly similar to an already approved biologic medication, known as the originator product (7, 8). They are designed to have no clinically meaningful differences in terms of biological activity, efficacy, safety, quality, and immunogenicity profile compared to the original biologic, allowing biosimilars to provide comparable therapeutic benefits while often being more cost-effective (7, 8).

The originator product with the active substance epoetin alfa is marketed under the brand name Eprex/Erypo (9). In 2007 several biosimilar products to Erypo were approved by the European Medicines Agency (EMA) and have since been marketed in Germany (10–13). These are available in a pre-filled syringe, with strengths ranging from 1,000 international units (IU) to 40,000 IU, excluding Abseamed, which is solely available up to a strength of 10,000 IU.

Despite the literature on the clinical experience of epoetin alfa biosimilars, key gaps remain in the economic analysis of these products. Previous studies have demonstrated therapeutic equivalence and safety to reference medicine (14–16), but have neglected critical aspects of cost savings to the German healthcare system. Addressing this gap, this study aimed to exclusively investigate the economic impact of the introduction of epoetin alfa biosimilars in the German SHI market using real-world data on unit volumes and list prices. Such analysis is essential to inform evidence-based healthcare policy and reimbursement decisions.

## 2 Methods

### 2.1 Study design

This budget impact analysis employed a retrospective database to assess the economic impact of the introduction of epoetin alfa biosimilars in the German SHI market. It is based on real-world data collected from January 2008 to December 2023 (16 years), focusing on price trends and volume usage to determine cost-savings and market dynamics.

### 2.2 Data sources

The primary data sources comprise price and product data from ABDATA and volume data from IQVIA Pharmascope, ensuring comprehensive coverage and reliability, as well as consistency in data collection and cost estimations. Price and product data from the ABDATA database provide detailed information on product identifiers (Pharmazentralnummer, PZN), strength, dosage, and pack size, along with official list prices of drugs sold in Germany. This dataset is updated on the 1st and 15th of each month. This analysis encompasses data from January 1st, 2007 to December 15th, 2023, with pricing data from 2007 used exclusively for the purpose of list price comparison with the originator product. Volume data, sourced from IQVIA Pharmascope, provides monthly sell-out units per individual PZN. For the current analysis, data from January 2008 until December 2023 were utilized. The ABDATA dataset represents the complete population of all available PZNs marketed in Germany during the study period. IQVIA Pharmascope data was matched to ABDATA data via the unique identifier PZN on monthly basis. If a PZN was missing in IQVIA Pharmascope data set for a specific period, the respective unit value was set to zero.

Neither ABDATA nor IQVIA’s Pharmascope are publicly accessible; they are subscription-based, professional databases and analytical services designed for the pharmaceutical industry.

### 2.3 Inclusion and exclusion criteria

The analysis included all listed Pharmaceutical Central Numbers (PZNs) for epoetin alfa products, encompassing both originator and biosimilar formulations. These were categorized according to their therapeutic indication and dosage strength, with nephrological strengths ranging from 1,000 IU to 10,000 IU and oncological strengths ranging from 20,000 IU to 40,000 IU. If a PZN was discontinued, it remained included as long as it appeared in the ABDATA dataset.

Products were excluded based on specific criteria. Epoetin variants such as epoetin beta, theta, and zeta were omitted from the cost-saving analysis due to regulatory or chemical differences. In addition, PZNs with an ex-factory price of zero were excluded, as these represented hospital-use or sample packages.

### 2.4 Calculations and analysis

The pricing structure consists of two key components: Firstly, the ex-factory price (“Abgabepreis des pharmazeutischen Unternehmens,” APU), representing the official list price, is published and accessible for each pharmaceutical product (identified by its PZN) on two specific dates per month, namely the 1st and 15th. In order to compile a single data set per each month, an average APU (AVG APU) is calculated per PZN. The AVG APU is calculated as the mean of the APU values available on the 1st and 15th of the month, expressed as:


$$\mathrm{AVG}\;\mathrm{AP}U_{\text{MONTH}}\;(\mathrm{per}\;\mathrm{PZN})=(\mathrm{AP}U_{1\mathrm{st}}+\mathrm{AP}U_{15\mathrm{th}})/2$$

In case a PZN was launched on the 15th, the AVG APU for that respective month will be equal to the APU of the 15th.

The second component is a price baseline (APU_fixed), representing the price level before the introduction of epoetin alfa biosimilars to the pharmaceutical market. APU_fixed is an average of all PZNs (originator and importers) with identical strengths and pack sizes available on April 1st, 2007, thus yielding a single price per package category defined by strength and pack size. Prices from importers are included to account for price competition prior to market entry of biosimilars.


$$\mathrm{APU}\_\text{fixed}=\frac{\left(\mathrm{AP}U_{\text{Originator}}+\mathrm{AP}U_{\text{ImporterA}}+\ldots +\mathrm{AP}U_{\text{ImporterX}}\right)}{n_{\text{Manufacturer}}}$$

Calculation of APU_fixed package per dosage and pack size combination based on the available APU prior to epoetin alfa biosimilars (cut-off date, April 1st, 2007).

In addition to price calculations, market share analyses were performed. Market shares were calculated based on volume data obtained by IQVIA Pharmascope, which provides monthly data at the PZN level, expressed in units (packages). The basis for determining the market share of epoetin alfa among epoetin derivates was the accumulated volume data per year for epoetin alfa, epoetin beta, epoetin theta and epoetin zeta. Market share calculations of specific dosages were based on volume data per year for epoetin alfa exclusively.

When discussing costs relevant for the German SHI system, the pharmacy selling price would seem to constitute an appropriate basis for drug cost calculation basis, but only after deducting mandatory manufacturing rebates, pharmacy rebates and rebates from confidential contracts with state insurance companies. Mandatory pharmacy as well as manufacturing rebates are defined in social legislation (§130 und §130a SGB V), while rebate contracts are individually negotiated by insurance companies. Pharmaceutical companies participating in such contracts are obliged to grant refunds according to the defined rebate conditions. These conditions vary across contract frameworks of the insurance company and are not publicly disclosed. The pharmacy selling price itself is determined through formal calculation rules defined within the Pharmaceutical Price Regulation (“Arzneimittelpreisverordnung” (17)). Given that the pharmacy selling price is influenced by legal regulations, changes at this level may occur independently of manufacturer price adjustments.

In order to circumvent the potential distortions caused by regulatory changes and the confidentiality of rebate contracts, the ex-factory price (APU) was utilized as the basis for cost-saving analyses, instead of the pharmacy selling price. Consequently, the identified cost savings in absolute numbers may be lower than the actual savings.

### 2.5 Market value and cost-savings

The market value of epoetin alfa is calculated using two approaches: fixed prices (APU_fixed) and list prices (AVG APU). These calculations use the following formulas:


$$\text{Market Value}\;(\mathrm{APU}\_\text{fixed})=\sum_{t=2008}^{2023}(\text{Volum}e_{\mathrm{PZN},t}*\mathrm{APU}\_\text{fixe}d_{\text{package}})$$

And


$$\begin{array}{l} \text{Market Value}\;(\text{list price}\_\mathrm{APU})=\\ \;\;\sum_{t=2008}^{2023}(\text{Volum}e_{\mathrm{PZN},t}*\mathrm{AVG}\;\mathrm{AP}U_{\mathrm{PZN},t}) \end{array}$$

Cost savings were determined by calculating the differential between the market value derived from list price calculation (list price_APU) and the market value based on the price baseline prior to the introduction of respective biosimilar products to the market (market value APU_fixed) using the formula:


$$\begin{array}{l} \text{Cost savings}=\text{Market Value}\;(\mathrm{APU}\_\text{fixed})\\ -\text{Market Value}\;(\text{list price}\;\mathrm{APU}) \end{array}$$

### 2.6 Statistical analysis

We calculated annual means and percentage changes to assess trends in pricing and volume, yielding a concise illustration of market trends, price fluctuations and the uptake of biosimilars over the investigated study period (2008–2023). AVG APU (month) was calculated per individual PZN and is an average value. APU_fixed, on the other hand, describes the average of all packages available on April 1st, 2007. The actual cost-savings analyses are calculated as the sum of the PZNs listed according to ABDATA for the analysis period. The exclusion of regulatory effects and confidential discounts was necessitated by two factors: firstly, the imperative of ensuring the comparability of the data; secondly, the recognition that confidential discounts are typically negotiated on an individual basis and are not available to the public. Assumptions were applied for the purpose of consistency, such as treating missing data within IQVIA Pharmascope as zero sales. Data extraction and integration were performed using ABDATA and IQVIA tools.

## 3 Results

### 3.1 Epoetin alfa market share analysis

The range of epoetin alfa preparations available in the German market following their regulatory approval within Europe in 2007 is presented in Table 1. In order to assess the impact of epoetin alfa within the market, we analysed annual market shares (2008–2023) of epoetin alfa in comparison to other epoetin derivatives, including epoetin beta, theta, and zeta, based on unit volume (number of packs sold per year). The results, presented in Table 2, indicate that epoetin alfa maintained the largest market share, contributing to 64% of total unit volume in 2023 (on average 57% unit volume between 2008 and 2023). Over the observed period, epoetin alfa demonstrated a remarkable growth of 23% in unit volume (Table 2). In contrast, the market share of other epoetin derivatives (e.g., beta, zeta and theta) exhibited a significant decline, with a cumulative reduction of 10%, indicating an increased adoption for epoetin alfa. The overall change in unit volume for all epoetin derivatives was +9% in 2023 compared to 2008.

**Table 1:** Epoetin alfa biosimilars available in the German market.

Preparation	Active ingredient	Approval holder	Active ingredient manufacturer	Cell lines	Market entry*
Originator product					
Erypo	Epoetin alfa	Janssen-Cilag	Johnson & Johnson	CHO-cells	Jun 1989
Biosimilars					
Epoetin alfa Hexal	Epoetin alfa	Hexal AG	Lek, Rentschler	CHO-cells	October 01, 2007
Binocrit	Epoetin alfa	Hexal AG	Lek, Rentschler	CHO-cells	October 15, 2007
Abseamed	Epoetin alfa	Medice Arzneimittel Pütter GmbH & Co. KG	Lek, Rentschler	CHO-cells	October 01, 2007
Retacrit	Epoetin zeta	Pfizer Pharma GmbH	Norbitec	CHO-cells	February 01, 2008
Silapo	Epoetin zeta	Stadapharm GmbH	Norbitec	CHO-cells	February 01, 2008
Neorecormon	Epoetin beta	Roche Pharma AG	Roche Diagnostics GmbH	CHO-cells	Jul 1997
Eporatio	Epoetin theta	Ratiopharm GmbH	Merckle Biotec GmbH	CHO-cells	February 01, 2010

*First product listing in official price and product database (e.g., Lauer-Taxe). CHO, Chinese hamster ovary.

**Table 2:** German market volume in units and market share (%) for epoetin derivatives.

Year	Epoetin alfa	Epoetin beta	Epoetin zeta	Epoetin theta	Total epoetin
2008	298,226 (57)	206,932 (39)	22,334 (4)	0 (0)	527,492
2009	250,394 (55)	153,361 (34)	49,186 (11)	0 (0)	452,941
2010	188,260 (52)	106,450 (29)	58,126 (16)	12,660 (3)	365,496
2011	172,941 (51)	82,973 (24)	62,981(18)	22,383 (7)	341,278
2012	164,358 (49)	74,723 (22)	67,619 (20)	26,554 (8)	333,254
2013	173,229 (52)	62,595 (19)	72,088 (22)	22,611 (7)	330,523
2014	183,416 (54)	55,872 (16)	83,358 (25)	16,289 (5)	338,935
2015	189,409 (54)	51,696 (15)	96,423 (27)	13,849 (4)	351,377
2016	201,542 (55)	51,235 (14)	103,782 (28)	13,101 (4)	369,660
2017	221,525 (56)	50,098 (13)	111,876 (28)	13,422 (3)	396,921
2018	258,905 (58)	47,977 (11)	126,726 (29)	10,918 (2)	444,526
2019	285,916 (60)	45,253 (10)	136,207 (29)	8,817 (2)	476,193
2020	311,362 (61)	41,948 (8)	150,307 (29)	8,005 (2)	511,622
2021	331,269 (62)	39,885 (7)	155,969 (29)	7,060 (1)	534,183
2022	347,513 (63)	39,466 (7)	158,657 (29)	7,074 (1)	552,710
2023	368,219 (64)	37,618 (7)	161,720 (28)	6,634 (1)	574,191
Total	3,946,484	1,148,082	1,617,359	189,377	6,901,302
Average 2008–2023 in %	57	17	23	3	
Delta units 2023 vs. 2008 in %	23	−10			
	9				

Data is given in units SHI (%). SHI, statutory health insurance.

During the investigated time period, a dramatic shift from originator products to epoetin biosimilar consumption was recorded, with biosimilar market shares rising from 42% in 2008 to 92% in 2023 (Supplementary Table S1).

### 3.2 Usage trends of epoetin alfa based on strength between 2008 and 2023

Building on these findings, a detailed market analysis of epoetin alfa usage across different nephrological (1,000 IU to 10,000 IU) and oncological strengths (20,000 IU to 40,000 IU) was undertaken to ascertain market trends (Table 3). The analysis revealed an average market share of 93% in 2023 (95% accumulated from 2008 to 2023) for nephrological strengths. Furthermore, distinct patterns of adoption over time were identified, providing valuable insights into the dynamics of market utilization. Between 2008 and 2023, the nephrological strengths of 4,000 IU and 2,000 IU were observed to have the highest utilization rates (27 and 20% market share in 2023, respectively, within the nephrological strength segment). Among these, epoetin alfa 4,000 IU showed a significant growth in unit volume (12%), indicating an increasing preference in clinical practice. In contrast, epoetin alfa 2,000 IU exhibited a decline in unit volume of 24%. The remaining strengths demonstrated consistent usage patterns.

**Table 3:** Market development of epoetin alfa (unit volume).

Year	Strength								Nephrological strength	Oncological strength
	1,000 IU	2000 IU	3,000 IU	4,000 IU	5,000 IU	6,000 IU	8,000 IU	10,000 IU	Sum 1,000–10,000 IU	Sum 20,000–40,000 IU
2008	23,313	88,810	39,127	83,010	17,697	18,932	10,492	8,393	289,774	8,452
2009	19,803	70,765	33,807	67,785	16,479	16,905	9,313	8,001	242,858	7,536
2010	14,437	53,056	24,561	51,746	12,063	12,819	6,972	6,072	181,726	6,534
2011	13,389	49,780	21,228	47,222	10,379	12,414	6,696	5,457	166,565	6,376
2012	12,219	47,740	20,971	43,558	9,870	12,490	5,668	4,823	157,339	7,019
2013	12,333	50,486	22,607	43,660	11,907	13,189	5,895	5,335	165,412	7,817
2014	10,900	52,059	23,177	47,635	13,145	14,371	6,740	5,901	173,928	9,488
2015	9,955	53,536	23,543	48,374	13,170	15,221	7,853	7,010	178,662	10,747
2016	10,188	55,875	24,321	51,692	14,008	17,231	9,269	8,065	190,649	10,893
2017	11,283	56,064	27,165	59,431	13,949	20,530	11,445	8,899	208,766	12,759
2018	12,461	61,434	31,657	68,391	15,779	24,938	15,020	11,399	241,079	17,826
2019	12,751	63,372	36,383	74,503	18,221	29,265	18,112	14,245	266,852	19,064
2020	13,306	66,612	39,679	79,237	19,515	34,461	22,049	16,487	291,346	20,016
2021	12,866	67,103	40,293	84,580	21,110	38,781	25,589	18,575	308,897	22,372
2022	12,355	65,924	43,195	88,537	23,190	43,163	27,086	20,557	324,007	23,506
2023	12,445	67,395	46,618	93,350	24,649	45,360	31,127	21,700	342,644	25,575
Delta in % 2023 vs. 2008	−47	−24	19	12	39	140	197	159		
Market share in 2023 (total) in %	3	18	13	25	7	12	8	6	93	7
Market share in 2023 (nephrological strengths in %)	4	20	14	27	7	13	9	6		

IU, international units.

### 3.3 Cost savings and economic impact of epoetin alfa biosimilars

**Figure 1:**
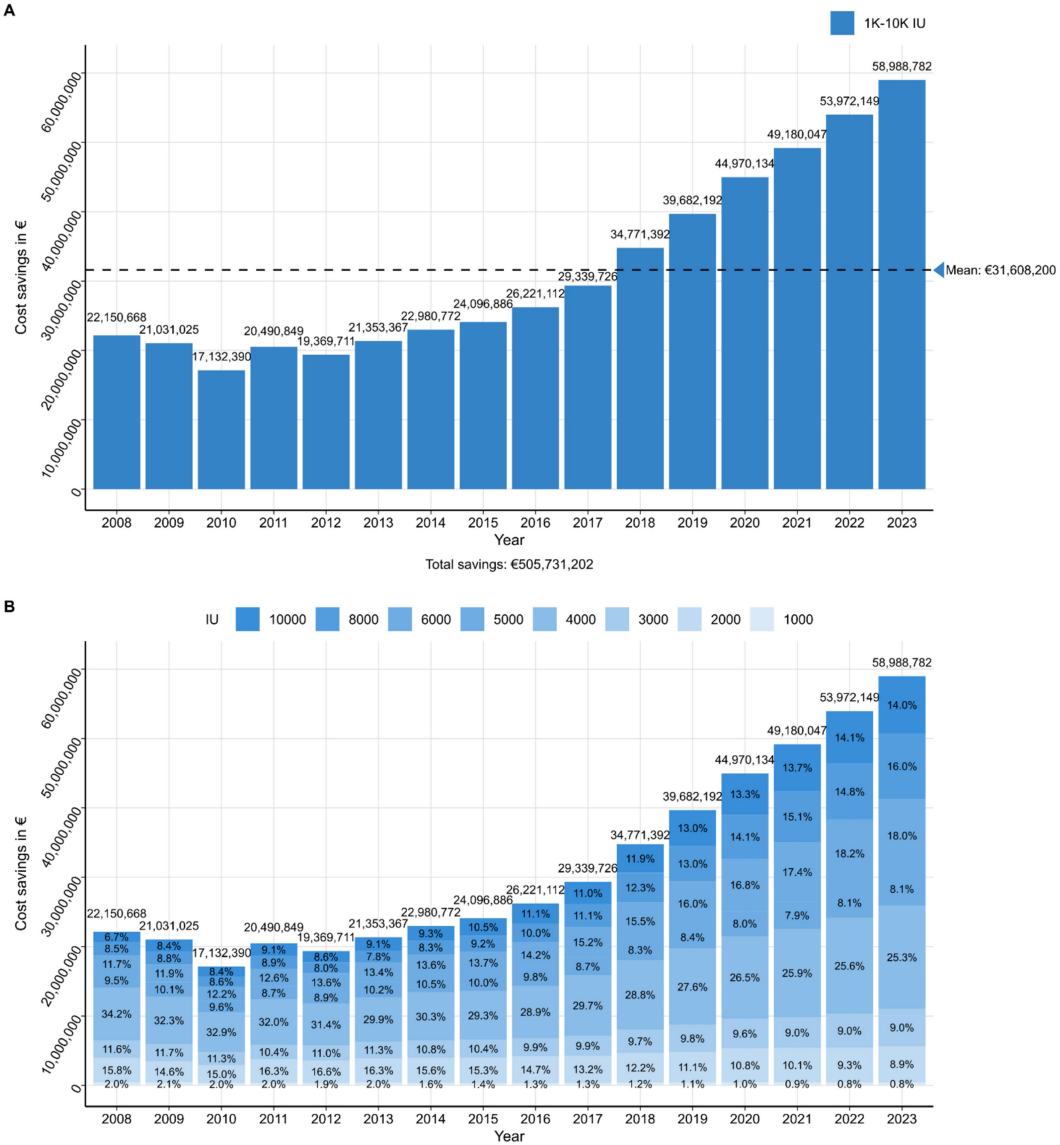
Year-wise cost savings on German statutory health insurance for epoetin alfa (1,000–10,000 IU) comparing market value (APU_fixed) and market value (list price_APU). APU, Abgabepreis pharmazeutisches Unternehmen; IU, international units.

These utilization patterns are closely linked to the economic implications of epoetin alfa adoption, particularly following the introduction of biosimilar epoetin alfa in 2007, the APU decreased consistently over the subsequent 6 years (2007–2012) for all available products, achieving a significant reduction of 47% (Table 4). From 2013 onwards, the price differences between the originator product and epoetin alfa biosimilars diminished, also due to adaptations of a fixed reference price. The relevant fixed reference price group encloses drugs to treat anemia and includes the molecules Darbepoetin, Erythropoetin (alfa, beta, theta, zeta) and PEG-Erythropoetin (Methoxy-Polyethylenglycol-Epoetin beta). These price developments resulted in cumulative cost-savings of €505,731,202 for the German SHI, encompassing all nephrological strengths of epoetin alfa between 2008 and 2023 (Figure 1; Supplementary Table S2).

**Table 4:** Price development of epoetin alfa from 2007 to 2023: Average ex-factory prices in € of nephrological strengths (1.000–10.000 IU) per year.

Year	Erypo (Janssen) in €	Epoetin alfa Hexal (Hexal) in €	Abseamed (Medice) in €	Binocrit (Sandoz/Hexal) in €	Delta in % Erypo vs. lowest priced biosimilar	Price decrease in % Erypo vs. 2007
2007	345.84	242.23*	251.12*	242.22*	−30	
2008	287.29	213.61	215.56	213.61	−26	−17
2009	287.39	210.80	210.80	210.80	−27	−17
2010	272.70	208.37	209.93	208.37	−24	−21
2011	206.42	184.32	184.98	184.32	−11	−40
2012	204.41	184.13	184.13	184.13	−10	−41
2013	182.21	182.19	182.18	182.19	0	−47
2014	182.21	182.21	182.21	182.21	0	−47
2015	182.21	182.21	182.21	182.21	0	−47
2016	182.21	182.21	182.21	182.21	0	−47
2017	182.21	182.21	182.21	182.21	0	−47
2018	182.21	182.21	182.21	182.21	0	−47
2019	182.21	182.21	182.21	182.21	0	−47
2020	181.16	181.15	181.16	181.15	0	−48
2021	180.12	180.10	180.12	180.10	0	−48
2022	176.30	176.29	176.30	176.29	0	−49
2023	172.48	172.48	172.48	172.48	0	−50

*Starting October 2007. IU, international units.

### 3.4 Potency-specific contributions to cost savings

To further delineate the sources of these economic effects, an analysis of cost savings per drug strength revealed that the nephrological strength of a 4,000 IU formulation of epoetin alfa contributed the most to overall savings, amounting to €143,720,363 and corresponding to 28% savings from 2008 to 2023 (Table 5). The disproportionately high contribution of the strength of 4,000 IU reflects its dominant role in clinical practice and the associated economic benefits stemming from its widespread adoption.

**Table 5:** Total cost-savings of epoetin alfa after biosimilar market entry (2008–2023).

Strength in IU	Savings in €	Savings in %	Savings in % *Base total epoetin alfa*
Nephrology			
1,000	6,456,207	1	1
2000	62,909,879	12	10
3,000	50,397,368	10	8
4,000	143,720,363	28	22
5,000	44,416,854	9	7
6,000	78,171,412	15	12
8,000	60,815,383	12	10
10,000	58,843,735	12	9
Oncology			
20,000	14,558,984	11	2
30,000	35,758,530	27	6
40,000	82,723,532	62	13

IU, international units.

A comparison of the cost saving effects of defined nephrological strengths and oncological strengths revealed that nephrological strengths account with a share of 79% of total savings, representing the largest impact.

## 4 Discussion

### 4.1 Background, key findings and interpretation

The 8th iteration of the ‘Impact of Biosimilar Competition in Europe‘(IQVIA observation 2022) has demonstrated a notable increase in the utilization of biosimilar products and market competition in Europe during the present decade (18). Biosimilars play a vital role in the European healthcare system, helping to manage healthcare costs by generating savings for payers, providing room for innovation, and increasing patient access to biologic therapies (18). In Germany, the biopharmaceutical market has demonstrated robust growth in gross sales since 2006 with biosimilar products inducing the highest market share (19).

Our analysis of unit volume data from 2008 to 2023 of epoetin alfa biosimilars from ABDATA and IQVIA Pharmascope databases is the first analysis investigating the economic impact of the introduction of epoetin alfa biosimilars to the German pharmaceutical market. This analysis revealed that the epoetin alfa products have the highest market share compared to other epoetin derivatives, indicating the highest preference and utilization in Germany. To the best of our knowledge, we are the first to report the overtime improvement of market dynamics of epoetin alfa derivatives in the German healthcare system. We observed a significant price development, with a robust decline in the ex-factory price of overall epoetin in nephrological strengths over a period of 6 years (2007 to 2012). Over 15 years this has been converted to a cumulative cost-saving of €505,731,202 for the German SHI. Such financial relief is attributed to increased competition among biosimilars, which drives prices down and enhances access to essential therapies for patients.

### 4.2 Comparison with previous studies

A similar cost-saving analysis on epoetin alfa biosimilars in European Union G5 countries by Aapro *et al.* revealed that the average treatment costs associated with epoetin alfa biosimilars are significantly lower than those of the originator products. Their study showed that the costs over a 15-week treatment period amounted to €4,643 for a dose of 30,000 IU and €6,178 for 40,000 IU of biosimilar epoetin alfa. In contrast, treatment with the originator epoetin alfa averaged around €7,168, while epoetin beta and darbepoetin alfa incurred higher costs of €7,389 and €8,299 (once weekly) or €9,221 (once every 3 weeks), respectively (20). Furthermore, Hörbrand *et al.* reported on cost savings of €2.23 per defined daily dose when erythropoiesis stimulating agent (ESA) biosimilars were preferred over originator products in patients suffering from renal anemia (21). A population-based study in a comparable patient population reported similar consumption rates of biosimilar and originator ESAs (22), which appears to contrast our findings. Nevertheless, despite the equivalent utilization of ESA biosimilars and originator products observed by Hörbrand et al., particularly within the nephrological setting, the demonstrated economic benefit of prescribing biosimilars over originator products or other treatment options underscores our findings regarding epoetin alfa.

### 4.3 Broader economic implications and policy perspectives

As market dynamics continue to evolve, it is anticipated that further cost reductions will emerge as biosimilars gain broader acceptance and utilization within healthcare systems (18, 19). Competitive pricing strategies employed by manufacturers, alongside potential rebates and discounts defined by healthcare providers within open house contracts, are likely to contribute to sustained economic benefits over time.

In Germany, pricing developments in the ESA market were also shaped by the revision of the fixed reference price in 2013, including epoetin alfa, epoetin beta, epoetin theta, epoetin zeta, PEG-Erythropoetin and Darbepoetin. Under this system, the reference price is determined by list price differentials and the market share of individual suppliers—meaning that a higher market share of lower-priced products leads to a lower overall reimbursement level. To avoid additional patient co-payments, manufacturers with higher list prices must align their prices with the defined reimbursement level, which in turn drives further price reductions and additional cost savings for the healthcare system.

The utilization of epoetin alfa biosimilars in Germany indicates a significant progression in the delivery of cost-effective healthcare for patients requiring ESAs. The substantial cost-savings observed when comparing biosimilar treatment to originator products not only alleviate financial pressures on healthcare systems but also enhance patient access to necessary therapies. It is therefore essential to continue monitoring of biosimilar utilization and pricing strategies in order to maximize these economic advantages in the future.

### 4.4 Limitations

A major strength of this budget impact analysis is the incorporation of reliable data sources to support the cost-saving assessment. However, it should be noted that the calculations based on the ex-factory price do not reflect the actual costs within the German SHI system. A proportion of the market, notably in the dialysis sector, is procured through centralized purchasing mechanisms and invoiced outside the purview of drug price regulation (approximately half of the volume based on epoetin alfa). Therefore, the market for epoetin alfa biosimilars is generally underestimated within this study. Moreover, the use of ex-factory prices may not fully provide an adequate basis for cost calculation, since reimbursement also encompasses costs associated with trade levels, such as wholesalers and pharmacies. In addition, the estimation of cost savings did not account for possible price fluctuations prior biosimilar market entry. As historical price data for individual PZNs were only available from 2007 onwards, pre-biosimilar pricing dynamics could not be examined. Therefore, potential fluctuations in drug prices unrelated to biosimilar competition cannot be entirely excluded.

## 5 Conclusion

In conclusion, the introduction of epoetin alfa biosimilars has had a significant economic impact on the German healthcare system, as reflected in their sustained market uptake, strength-specific utilization patterns, and associated cost reductions since 2007. These findings highlight the contribution of biosimilar competition to price efficiency and the long-term sustainability of pharmaceutical expenditures within the statutory health insurance system. Moreover, the results underscore the policy relevance of biosimilars in supporting equitable access to biologic therapies and efficient allocation of healthcare resources.

## Data Availability

The data analyzed in this study is subject to the following licenses/restrictions: The data that has been analysed is not subject to public disclosure. However, these data were obtained from IQVIA and ABDATA by Sandoz/Hexal. Requests to access these datasets should be directed to Ilka Raitner, ilka.raitner@sandoz.com.
