# Supplementary material for: An analysis of cost-saving in the German statutory health insurance system due to the introduction of epoetin alfa biosimilars in Germany
Source: Front Public Health. 2025 Dec 16;13:1667315. doi: 10.3389/fpubh.2025.1667315 (PMC12748244; doi:10.3389/fpubh.2025.1667315)
Supplement: Supplementary file 1 [file Data_Sheet_1.docx]

# Supplementary Material

**Supplementary Table 1.** Market share of epoetin alfa biosimilars based on unit volume per year.

| **Year** | **Eprex** | **Erypo** | **Epoetin alfa Hexal** | **Abseamed** | **Binocrit** | **Market share biosimilars** |
| --- | --- | --- | --- | --- | --- | --- |
| **2008** | 29,278 | 142,409 | 70,915 | 37,613 | 18,011 | 42% |
| **2009** | 15,797 | 91,387 | 67,656 | 52,257 | 23,297 | 57% |
| **2010** | 8,401 | 59,940 | 56,307 | 46,199 | 17,413 | 64% |
| **2011** | 4,809 | 49,587 | 53,816 | 47,090 | 17,639 | 69% |
| **2012** | 4,281 | 43,964 | 52,098 | 49,371 | 14,644 | 71% |
| **2013** | 3,122 | 42,462 | 53,254 | 61,409 | 12,982 | 74% |
| **2014** | 4,728 | 37,890 | 60,769 | 67,480 | 12,549 | 77% |
| **2015** | 294 | 39,832 | 74,427 | 65,597 | 9,259 | 79% |
| **2016** | 0 | 37,950 | 85,373 | 69,633 | 8,586 | 81% |
| **2017** | 0 | 33,478 | 94,095 | 84,026 | 9,926 | 85% |
| **2018** | 0 | 34,250 | 119,177 | 93,129 | 12,349 | 87% |
| **2019** | 0 | 34,617 | 133,738 | 97,079 | 20,482 | 88% |
| **2020** | 0 | 30,412 | 136,724 | 90,170 | 54,056 | 90% |
| **2021** | 0 | 29,415 | 153,989 | 91,394 | 56,471 | 91% |
| **2022** | 0 | 29,529 | 161,495 | 93,478 | 63,011 | 92% |
| **2023** | 0 | 28,106 | 166,456 | 100,669 | 72,988 | 92% |

**Supplementary Table 2.** Year-wise cost-savings on German statutory health insurance of epoetin alfa by year and strength.

| **Year** | **IU** | | | | | | | |
| --- | --- | --- | --- | --- | --- | --- | --- | --- |
|  | **1000** | **2000** | **3000** | **4000** | **5000** | **6000** | **8000** | **10000** |
| **2007** | 36,440 | 404,883 | 367,538 | 1,236,711 | 273,882 | 330,196 | 214,128 | 227,114 |
| **2008** | 441,044 | 3,508,982 | 2,570,235 | 7,577,686 | 2,093,769 | 2,599,779 | 1,885,555 | 1,473,618 |
| **2009** | 431,576 | 3,076,335 | 2,467,248 | 6,801,900 | 2,123,209 | 2,507,070 | 1,846,604 | 1,777,084 |
| **2010** | 338,084 | 2,576,514 | 1,941,680 | 5,639,480 | 1,644,602 | 2,086,796 | 1,466,440 | 1,438,794 |
| **2011** | 401,883 | 3,338,720 | 2,139,082 | 6,554,077 | 1,792,021 | 2,587,460 | 1,814,245 | 1,863,360 |
| **2012** | 372,165 | 3,219,320 | 2,136,712 | 6,083,883 | 1,717,997 | 2,628,715 | 1,548,603 | 1,662,317 |
| **2013** | 418,834 | 3,485,796 | 2,415,416 | 6,382,284 | 2,183,953 | 2,860,654 | 1,673,373 | 1,933,057 |
| **2014** | 370,172 | 3,589,998 | 2,476,240 | 6,967,273 | 2,410,620 | 3,115,908 | 1,912,421 | 2,138,140 |
| **2015** | 338,027 | 3,688,819 | 2,515,247 | 7,071,375 | 2,415,181 | 3,300,153 | 2,228,116 | 2,539,968 |
| **2016** | 345,934 | 3,851,348 | 2,598,827 | 7,568,399 | 2,568,855 | 3,735,939 | 2,629,876 | 2,921,935 |

Legend: IU, international units
